# Ошибки: Клинические и молекулярно-генетические характеристики пациентов с нарушением формирования пола 46,XY, обусловленным мутациями в гене NR5A1. [Проблемы эндокринологии 2020;66(3):62-69. doi: 10.14341/probl12445]

**DOI:** 10.14341/probl12848

**Published:** 2021-12-30

**Authors:** Н. Ю. Калинченко, А. А. Колодкина, Н. Ю. Райгородская, А. Н. Тюльпаков

**Affiliations:** Национальный медицинский исследовательский центр эндокринологии; Национальный медицинский исследовательский центр эндокринологии; Саратовский государственный медицинский университет им. В.И. Разумовского; Национальный медицинский исследовательский центр эндокринологии

**Keywords:** нарушение формирования пола, гипоспадия, стероидогенный фактор, NR5A1, дисгенезия

## Abstract

В статье «Клинические и молекулярно-генетические характеристики пациентов с нарушением формирования пола 46,XY, обусловленным мутациями в гене NR5A1», опубликованной в Т. 66 № 3 журнала «Проблемы Эндокринологии» за 2020 г. (doi: 10.14341/probl12445), допущены ошибки:

- в аннотации «Среди обнаруженных мутаций 15 ранее описаны не были» была исправлена информация на «Среди обнаруженных мутаций 22 ранее описаны не были»;

- в подразделе РЕЗУЛЬТАТЫ «У 36 пациентов было выявлено 31 вариант в гене NR5A1, 15 из которых ранее описаны не были» внесены изменения на «У 36 пациентов было выявлено 31 вариант в гене NR5A1, 22 из которых ранее описаны не были»;

- в подразделе РЕЗУЛЬТАТЫ «Среди впервые выявленных вариантов изменений в гене NR1A1 два приводят к образованию стоп-кодона – p.Y197X и p.Y25X, два к сдвигу рамки считывания — p.N385SfsX10 и p.L245AfsX53, что не позволяет сомневаться в их патогенности. Среди ранее неописанных вариантных изменений 5 миссенсмутаций (p.C283Y, p.С283F, p.H24Q, p.M126K, p.A82T) и 1 синонимичная замена, затрагивающая сайт сплайсинга (E330E), были оценены как патогенные, а 5 других — как вероятно патогенные» была исправлена информация на «Среди впервые выявленных вариантов изменений в гене NR1A1 два приводят к образованию стоп-кодона — p.Y197X и p.Y25X, два к сдвигу рамки считывания — p.N385SfsX10 и p.L245AfsX53, что не позволяет сомневаться в их патогенности. Среди ранее неописанных вариантных изменений 5 миссенс-мутаций (p.C283Y, p.С283F, p.H24Q, p.M126K, p.A82T ) и 1 синонимичная замена, затрагивающая сайт сплайсинга (E330E), были оценены как патогенные, а 5 других — как вероятно патогенные»;

- внесены исправления в таблицу 2. Приведенная ранее информация в статье не должна была сколь-нибудь существенно отразиться на восприятии информации читателями и/или интерпретации представленных данных. Авторы выражают сожаления за неверную информацию в опубликованной ранее статье.

## Abstract (en)

n the article some corrections were needed. Abstract: “Heterozygous SF1 variants were found in 36 out of 310 (11.6%) of cases, among them 15 were not previously described”. has been corrected to read “Heterozygous SF1 variants were found in 36 out of 310 (11.6%) of cases, among them 22 were not previously described”. Results: “Heterozygous SF1 variants were found in 36 out of 310 (11.6%) of cases, among them 15 were not previously described”, has been corrected to read “Heterozygous SF1 variants were found in 36 out of 310 (11.6%) of cases, among them 22 were not previously described”. Among the newly identified variants in the NR1A1 gene, two lead to the premature stop codon -p. Y197X and p. Y25X, two lead to a shift in the reading frame-p. N385fs and p. L245fs, which does not allow us to doubt their pathogenicityAmong the previously undescribed variant changes, 5 missense mutations (p. C283Y, p. C283B, p.H24Q, p.M126K, p.E81K) and 1  synonymous substitution affecting the splicing site (E330E) were evaluated as pathogenic, and 5 others as probably pathogenic.

Has been corrected to read: Among the newly identified variants in the NR1A1 gene, two lead to the premature stop codon -p. Y197X and p. Y25X, two lead to a shift in the reading frame — p.N385SfsX10 and p.L245AfsX53, which does not allow us to doubt their pathogenicity Among the previously undescribed variants, 5 missense mutations (p.C283Y, p.С283F, p.H24Q, p.M126K, p.A82T) and 1 synonymous substitution affecting the splicing site (E330E) were predicted as pathogenic, and 5 others as probably pathogenic by calculating pathogenicity. The authors apologize for these errors.

**Table:** Таблица 2. Фенотипические, гормональные и молекулярно-генетические характеристики пациентов. Примечания: Т – тестостерон; ЛГ — левая гонада; ПГ — правая гонада; МТ — малый таз; ЛПК — левосторонний паховый крипторхизм; ППК — правосторонний паховый крипторхизм; БГ — большая губа; * — патогенность определена для впервые выявленных вариантных изменений: П — патогенная мутация, ВП — вероятно патогенная, в скобках указаны коэффициенты (PP, PM и др.) для расчета патогенности [12, 13]; ж › м – смена пола с женского на мужской; АМГ >28 нг/мл — норма для мальчиков до 1 года [23].

1	ж	4 мес	III			10.6/2.2	1.3/7.59	12	c.del1273_1278p.E425_Vl426del	П (PS4, PP4, PM2, PVS1)
2	ж	11 мес	III	нет	нет	14/	10.6	13.7	c.1152_1153ins(25) p.N385SfsX10	П (PP4, PM2, PVS1)
3	ж	1 год	III	рудимент	ЛГ-МТ, ППК	6.72/0.21	0.36	17.6	c.671C>T:p.P224L	ВП (PS4, PM2, PP2, PP4)
4	ж	4 года	Ж	есть					c.104G>A:p.G35D	
5	ж	13 лет	II	рудимент					c.1025C>T:p.S342L	ВП (PS4, PM2, PP2, PP4)
6	ж	13 лет	II		БГ				c.1003A>C:p.T335P	ВП (PS4, PM2, PP2, PP4)
7	ж	3 мес	II	нет	ПК	3.93/1.22	1.3/13.3	41	c.848G>T:p.C283F	П (PS4, PM2, PM5, PP2, PP4)
8	ж	16 лет	II	нет	ПК	34/27.7	15.7		c.937C>T:p.R313C	
9	ж	1 г. 4 мес	III	нет	БГ	0.87/0.2	0.1	20	c.103-3C>A	
10	ж	10 мес	II	нет	ПК	3.6/1.5	0.22/24	40	c.70C>G:p.H24D	П (PS4, PM2, PM5, PP3, PM1, PP2, PP4)
11	ж	1 г. 5 мес	III	нет	ППК, ЛГ- мошонка	10.5/2.16	0.5/4.51		c.37T>C:p.C13R	
12	ж	15 лет	Ж		ПБК, ЛПК	85/43	9.5		c.72C>G:p.H24Q	П (PS4, PM2, PM5, PP2, PP4)
13	ж	5 лет	III	нет	ПК				c.251G>A p.R84H	
14	ж	4 мес	Ж	есть	БГ	7.0/0.5	3.17/4.89		c.102+1G>T	
15	ж	10 лет	II	нет	ПК	5.54/0.3	/7.45	8.45	c.106T>C:p.F36L	ВП (PS4, PM2, PP2, PP4)
16	ж	нет инф.	Ж						c.245-1G>T	
17	ж	нет инф.	III						c.848G>A:p.C283Y	П (PS4, PM2, PM5, PP2, PP4)
18	ж	10 лет	II	нет		10.3/3.2	10		c.728_733dupGCATCp.L245AfsX53	П (PP4, PM2, PVS1)
19	ж › м	1,5 мес	IV		ПК	7.2/4.2	3.1/10	18	c.1139-1G>T	
20	м	2 мес	IV			5.03/1.69	4.0/7.8		c.244G>A:p.A82T	П (PS4, PM2, PM5, PP2, PP4)
21	м	1 мес	III	нет	в мошонке	18.6/1.0	0.8	18.5	c.721C>T:p R241W	ВП (PS4, PM2, PP2, PP4)
22	м	4 г.	IV			1.64/0.1	0.09		c. 75C>A:p.Y25X	П (PP4, PM2, PVS1)
23	м	5 мес	IV		ППК, ЛГ- мошонка	5.0/2.2	5.6/33	44	c.937C>T:p.R313C	
24	м	1 мес	III		ПК		4.3		c.937C>T:p.R313C	
25	м	13 лет	III	нет	ППК, ЛГ- мошонка	14.4/5.32	15.9		c.T377A:p.M126K	П (PS4, PM2, PM5, PP2, PP4)
26	м	4 мес	IV		в мошонке	7.2/4.2	2.63		c.C86T:p.T29M	
27	м	10 лет	III	нет	ПК	1.7/0.2	0.26	31	c.591C>A:p.Y197X	П (PP4, PM2, PVS1)
28	м	1 г. 2 мес	III	нет	ПК	2.4/0.48	0.5/14		c.C909G:p.S303R	ВП (PM2, PP3, PM1, PP4)
29	м	15 лет	III	нет	ЛПК, ПГ — в мошонке	9.1/8.1	26.2	42	c.G938A:p.R313H	
30	м	18 лет	III	нет	ПК	8/5.5	18.3	0.6	c.251G>A:p.R84H	
31	м	12 мес	III	нет		1.1/0.2	0.2		c.1289G>A:p.S430N	ВП (PM2, PM5, PP3, PM1, PP4)
32	м	11 лет	III			2.64/0.19	0.27		c.962G>T:p.G321V	ВП (PM2, PP3, PM1, PP4)
33	м	5 мес	IV		ПК	2.3/0.2	0.03/4.6	60.4	c.251G>A:p.R84H	
34	м	18 лет	IV	нет	ППК, ЛГ — мошонка	9.6/6.9	11/20.8		c.951delC:p.H317QfsX17	П (PP4, PM2, PVS1)
35	м	16 лет	III	нет	ПК	8/5.5	15.4		c.951delC:p.H317QfsX17	П (PP4, PM2, PVS1)
36	м	нет инф.	III						c.990G>A:p.E330E	П (PM2 PVS1 PP4)

 

